# Variables Affecting Shoot Growth and Plantlet Recovery in Tissue Cultures of Drug-Type *Cannabis sativa* L.

**DOI:** 10.3389/fpls.2021.732344

**Published:** 2021-09-21

**Authors:** Janesse E. Holmes, Samantha Lung, Danielle Collyer, Zamir K. Punja

**Affiliations:** Department of Biological Sciences, Simon Fraser University, Burnaby, BC, Canada

**Keywords:** meristems, nodal explants, shoot growth, rooting, plantlet recovery, micropropagation, callogenesis, *Cannabis sativa* L.

## Abstract

Tissue culture approaches are widely used in crop plants for the purposes of micropropagation, regeneration of plants through organogenesis, obtaining pathogen-free plantlets from meristem culture, and developing genetically modified plants. In this research, we evaluated variables that can influence the success of shoot growth and plantlet production in tissue cultures of drug-type *Cannabis sativa* L. (marijuana). Various sterilization methods were tested to ensure shoot development from nodal explants by limiting the frequency of contaminating endophytes, which otherwise caused the death of explants. Seven commercially grown tetrahydrocannabinol (THC)-containing cannabis genotypes (strains) showed significant differences in response to shoot growth from meristems and nodal explants on Murashige and Skoog (MS) medium containing thidiazuron (1 μM) and naphthaleneacetic acid (0.5 μM) plus 1% activated charcoal. The effect of Driver and Kuniyuki Walnut (DKW) or MS basal salts in media on shoot length and leaf numbers from nodal explants was compared and showed genotype dependency with regard to the growth response. To obtain rooted plantlets, shoots from meristems and nodal explants of genotype Moby Dick were evaluated for rooting, following the addition of sodium metasilicate, silver nitrate, indole-3-butyric acid (IBA), kinetin, or 2,4-D. Sodium metasilicate improved the visual appearance of the foliage and improved the rate of rooting. Silver nitrate also promoted rooting. Following acclimatization, plantlet survival in hydroponic culture, peat plugs, and rockwool substrate was 57, 76, and 83%, respectively. The development of plantlets from meristems is described for the first time in *C. sativa* and has potential for obtaining pathogen-free plants. The callogenesis response of leaf explants of 11 genotypes on MS medium without activated charcoal was 35% to 100%, depending on the genotype; organogenesis was not observed. The success in recovery of plantlets from meristems and nodal explants is influenced by cannabis genotype, degree of endophytic contamination of the explants, and frequency of rooting. The procedures described here have potential applications for research and commercial utility to obtain plantlets in stage 1 tissue cultures of *C. sativa*.

## Introduction

*Cannabis sativa* L., a member of the Cannabaceae family, is a dioecious, annual flowering plant that has been cultivated for thousands of years for its fiber (as hemp) and medicinal and psychotropic properties (as cannabis or marijuana). Vegetative cuttings are the conventional method for commercial propagation of cannabis to ensure rapid propagation of desired genotypes without the introduction of genetic variability resulting from sexual reproduction as *C. sativa* is allogamous (Punja and Holmes, 2020). However, vegetative cuttings can lose vigor, since donor (mother or stock) plants can be affected by fungal pathogens and viruses that can reduce their growth and quality (Punja et al., 2019; Punja, 2021). However, maintaining donor plants to be used as a source of vegetative cuttings can be time-consuming and space-intensive. Hence, there is interest in using tissue culture methods to propagate cannabis, as it is recognized as a means to potentially increase plant numbers of desired genotypes (micropropagation), and maintain them in a controlled and stable environment (preservation) (Monthony et al., 2021). In addition, callus production (callogenesis) from explants in tissue culture can potentially be used to increase plant numbers through organogenesis (Page et al., 2021). However, there are still many challenges remaining in establishing a micropropagation and callusing system for cannabis plants (Monthony et al., 2021). The quality of source (donor) plants, genotype or strain used, surface sterilization methods, explant type, and tissue culture medium and growth regulators can all influence the success rate of recovery of plantlets in stage 1 of tissue culture (the introduction of explant material for establishment of cultures). Rooting and acclimatization of plantlets are challenging aspects to tissue culture of hemp and cannabis as well. Lata et al. (2009) reported successful propagation of strain MX-1 of cannabis in tissue culture, but it is not known whether this method can be applied to other genotypes of cannabis. Monthony et al. (2021) described a procedure for the micropropagation of six cannabis genotypes, but rooting and plantlet recovery were not addressed. The success of a tissue culture method for cannabis is contingent upon obtaining a high frequency of plantlets growing independently in growth media.

In this research, we investigated various factors that can influence the recovery of plantlets of cannabis in tissue culture. The objectives of this study were to (1) assess the efficacy of sterilization methods to reduce the frequency of contaminants originating from donor plants; (2) recover and identify the various microbes present as contaminants in tissue cultures; (3) evaluate shoot growth from meristems of five different genotypes; (4) determine the responses of seven different genotypes to shoot growth from nodal explants; (5) evaluate the response of 11 different genotypes to callus development; (6) develop a rooting method for plantlets derived from tissue culture; and (7) evaluate acclimatization responses in different growth substrates (peat, rockwool, and hydroponic system) to achieve a high frequency of plantlet recovery.

## Materials and Methods

### Genotypes

The genotypes used were Moby Dick (MBD), Space Queen (SPQ), Copenhagen Kush (CPH), Cheesequake (CHQ), Pennywise (PWE), Girl Scout Cookie (GSC), Death Bubba (DEB), Afghan Kush (AFK), Island Honey (ISH), Pink Kush (PNK), Pure CBD (CBD), and White Rhino (WHR). Various combinations of genotypes were used in experiments, depending on their availability. Donor plants of the various genotypes were grown by a licensed producer according to Health Canada requirements in a controlled-environment room or under commercial greenhouse conditions The growing medium for the donor plants was either pure coco fibers or a coco fiber:vermiculite mix (3:1). The plants in the controlled-environment room were placed under two Sunblaster brand 54-watt 6400 k T5HO lights with a 24-h photoperiod. The plants were watered as needed with a solution of 1 ml/L Sensi Grow Coco pH Perfect A+B (Advanced Nutrients, Los Angeles, CA, Untied States) and 1 ml/L CALiMAGIc (General Hydroponics, Santa Rosa, CA, United States) adjusted to a pH of 5.8–6.2 using pH-Down (Advanced Nutrients, Los Angeles, CA, Untied States) (Scott and Punja, 2021). The plants in the commercial greenhouse were grown according to the standards for the industry and kept under a 16-h photoperiod to maintain vegetative growth. Explants were taken as needed for the tissue culture experiments described.

### Explants

**Figure 1:**
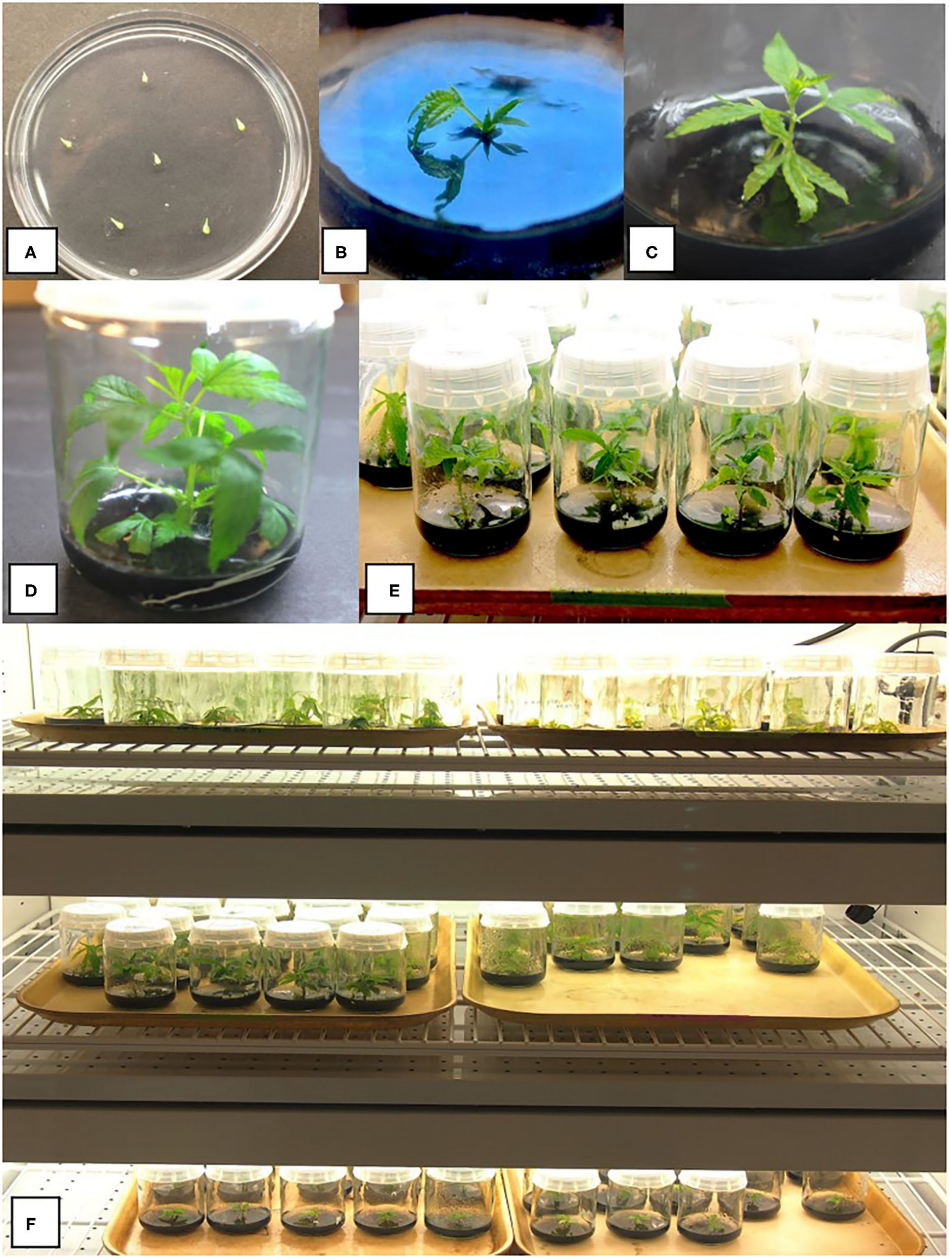
Various stages of growth of shoots derived from meristems of drug-type cannabis genotypes grown on a multiplication medium (MM) containing activated charcoal. **(A)** Meristem explants placed on an agar medium in a 90-mm Petri dish to show their small size. **(B)** Early shoot growth after 4 weeks in culture from a meristem. **(C)** Shoot growth after 8 weeks. **(D,E)** Shoot growth after 10 weeks from meristems. **(F)** Baby food jars containing meristem explants at different stages of development in a controlled environment growth chamber. A number of different strains are shown. Conditions of incubation are described in the Methods section.

**Figure 2:**
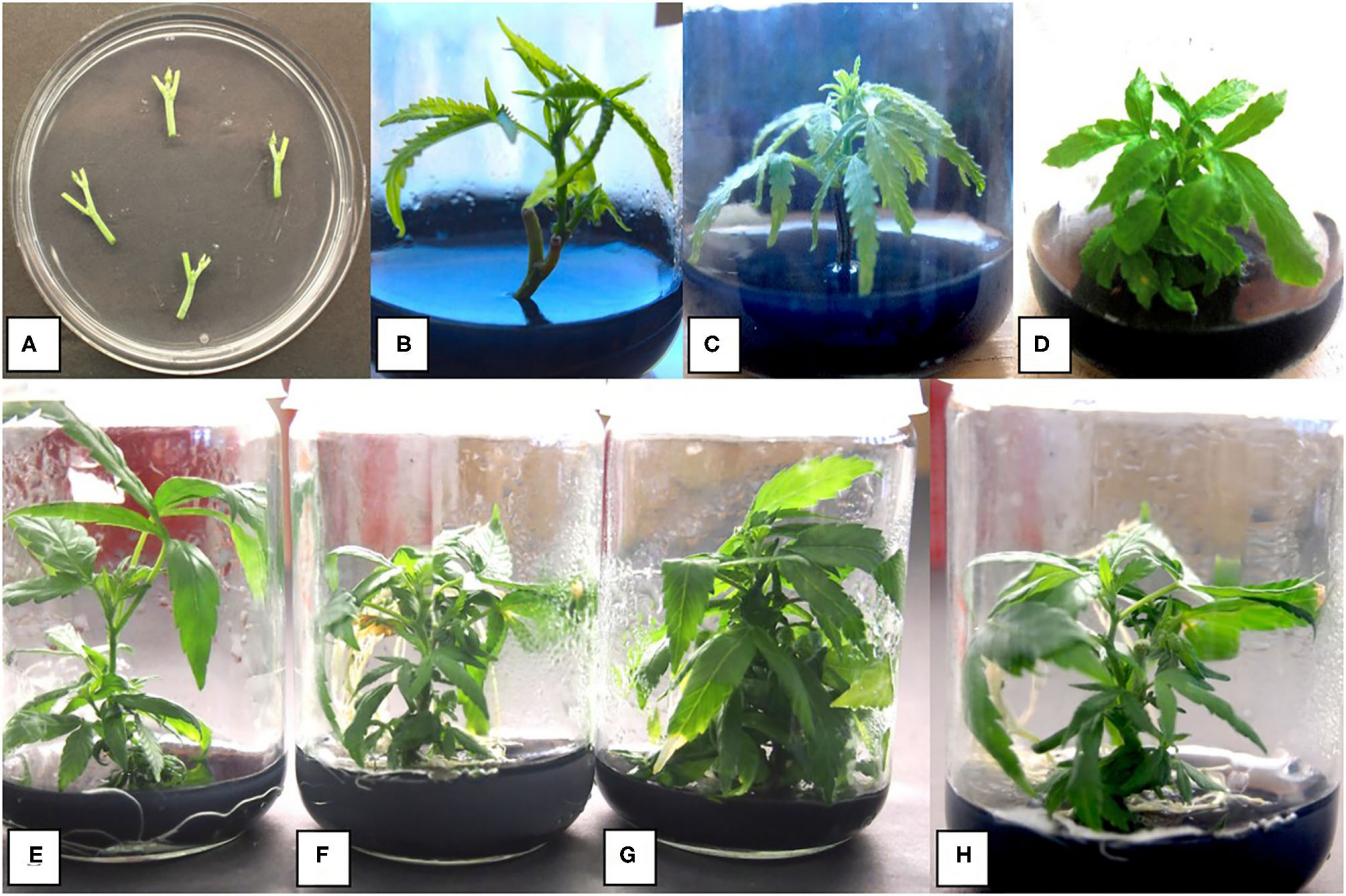
Stages of growth of shoots derived from nodal segments of drug-type cannabis genotypes grown on MM containing activated charcoal. **(A)** Nodal stem explants placed on an agar medium in a 90-mm Petri dish to show their size. **(B)** Shoot growth after 4 weeks from a nodal stem explant. The rate of growth is greater than that from a meristem. **(C)** Shoot growth after 6 weeks. **(D)** Shoot growth after 8 weeks. **(E)** Shoot growth after 8 weeks of genotype Death Bubba (DEB) from nodal explants. **(F)** Shoot growth after 8 weeks of genotype Pink Kush (PNK) from nodal explants. **(G)** Shoot growth after 8 weeks of genotype White Rhino (WHR) from nodal explants. **(H)** Shoot growth after 8 weeks of genotype Moby Dick (MBD) from nodal explants.

The explants tested included meristems, nodal segments, leaves, and petiole tissues. Meristems were dissected from terminal and lateral shoots from donor plants (Figure 1A). The external tissue was cut away with a scalpel, and two sets of primordial leaves were left to protect the meristem from excessive damage from sterilization. Nodal segments with axillary buds were removed from lateral stems of the plants and trimmed to a 1-cm length (Figure 2A). Leaves were trimmed from the plants and used for leaf explants in callus induction experiments. They were cut into pieces measuring 0.5 to 1 cm^2^. Petiole segments from young leaves were cut into 1-cm long pieces for callus induction.

### Sterilization

The standard sterilization protocol involved placing explants in a stainless steel tea strainer and immersing them in 70% EtOH in a glass beaker for 1 min while stirring with a magnetic stir bar. The explants were transferred to a 10% bleach solution (0.625% NaOCl) with 0.1% Tween 20 for 20 min, followed by three rinses in sterile distilled water, 3 min each. In some of the experiments, the bleach concentration, length of sterilization, and length of rinsing were adjusted according to explant type and source. The explants were blotted dry on sterile filter paper placed in a laminar flow hood and used immediately for tissue culture experiments.

### Media

The medium containing Murashige and Skoog basal salts, as described by Lata et al. (2009), was used in initial tissue culture experiments. The medium was supplemented with myo-inositol (0.1 g/L) and activated charcoal (1 g/L). The growth regulators added were thidiazuron (TDZ, 1 μM) and naphthaleneacetic acid (NAA, 0.5 μM). This combination of ingredients constitutes a multiplication medium, referred to as MM. All the chemical reagents were from Sigma-Aldrich (St. Louis, MO). The medium was adjusted to pH 6.6 ± 0.01 before autoclaving for 20 min at 121°C. Following autoclaving, the pH dropped to ~5.8. The medium was dispensed into 220 ml culture jars C1770 with Magenta B caps (Phytotechnology Laboratories®, Lenexa, KS, United States), and each jar received ~25 ml of the medium. A single meristem or nodal explant was placed inside each jar. For callus induction, MM without activated charcoal (MM-AC) was used. Each 90-mm Petri dish received ~25 ml of the medium onto which four to five leaves or petiole segments were placed.

### Meristem and Nodal Explant Growth

For meristems, genotypes Cheesequake, Pure CBD, Moby Dick, Pennywise, and Space Queen were used. Jars containing meristem explants prepared as described above were placed inside a Conviron A1000 growth chamber (Conviron Environments Ltd., Manitoba, Canada) under T5 fluorescent lights with an 18-h photoperiod and a light intensity of 102 μmoles m^−2^ s^−1^ (Figure 1). The temperature range was 25 ± 2°C. The meristems were left in culture for 6 weeks and transferred to fresh MM medium and incubated for another 4 weeks (Figure 1). At this time, shoot height, number of axillary buds developing, and number of shoots that formed were evaluated (Table 1). There was a minimum of 10 replicate jars, each containing a meristem for each genotype, and the experiment was conducted three times (*n* = 30) using different sources of explants of the same genotype. The shoots were maintained in culture for a maximum of 3 months by monthly transfer to fresh MM medium.

**Table 1:** Comparison of growth of shoots from meristems and nodal explants of five genotypes of drug-type *Cannabis sativa*.

**Explant type**							
**Meristems^a^**					**Nodal segments^a^**		
	**Shoot height (cm)**	**No. of buds**	**No. of shoots**		**Shoot height (cm)**	**No. of buds**	**No. of shoots**
**Genotype^b^**				**Genotype^b^**			
CBD	3.6 (0.37) a,d	8.0 (1.38) a	1.9 (0.32) a	BLD	4.9 (0.51) a	4.6 (0.4) a	0.4 (0.13) a
CHQ	1.5 (0.08) b,c	6.2 (0.69) a,c	0.5 (0.18) c	SWD	3.8 (0.27) a	3.8 (0.38) a,b	0.4 (0.17) a
MBD	4.5 (0.42) a	6.9 (0.84) a	1.9 (0.27) a	MBD	2.2 (0.27) b	2.9 (0.28) b	0.3 (0.11) a
SPQ	1.9 (0.15) b	3.4 (0.37) c	0.2 (0.07) b	SPQ	1.5 (0.11) c	1.2 (0.11) c	0.0 (0.0) b
PWE	3.1 (0.25) c,d	5.6 (0.53) a,c	2.0 (0.31) a	–	–	–	–

a *Data for meristems were collected after 10 weeks in culture and for nodal explants after 6 weeks in culture*.

b *Within each explant type, genotypes were compared with each other for shoot height, number of buds produced, and number of shoots. Data presented are from 10 explants, and the experiment was conducted three times (n = 30). Means were compared following ANOVA, and means separation was achieved by Tukey's honestly significant difference (HSD) test. Means within a column followed by the same letter are not significantly different at P < 0.05*.

For nodal segments, genotypes BLD, SPQ, MBD, and SWD were used. Jars containing a segment each were incubated under T5 fluorescent lights with an 18-h photoperiod and a light intensity of 102 μmoles m^−2^ s^−1^ and a temperature range of 21–27°C for 2 weeks. The shoots were transferred to fresh MM medium and allowed to grow for another 4 weeks (Figure 2). The percentage of explants surviving, shoot height, number of shoots per explant, number of buds developing, and number of leaves were recorded (Table 1). For each genotype tested, there was a minimum of 10 replicate jars, each containing a nodal explant, and the experiment was conducted three times (*n* = 30) using different explant sources of the same genotype. To determine if there were differences between the genotypes, the data were analyzed using Statplus version 2.21 and R systems (version 3.3.3). ANOVA was performed followed by Tukey's honestly significant difference (HSD) test to determine significance at *P* **< ** 0.05.

### Comparison of DKW and MS Salts

**Figure 3:**
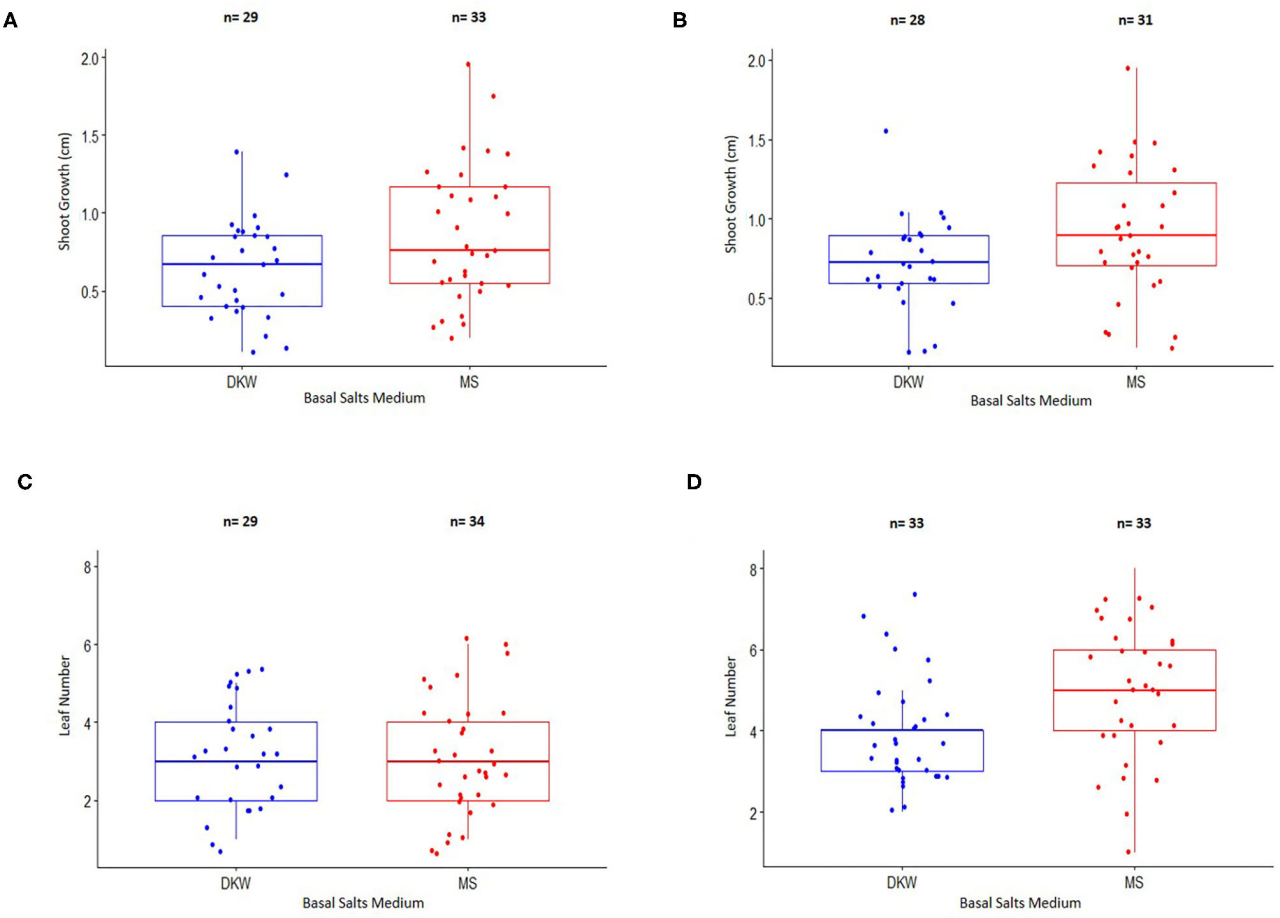
Comparison of shoot growth and leaf number from nodal stem explants of two cannabis genotypes placed on a basal salt medium containing either Driver and Kuniyuki Walnut (DKW) or Murashige and Skoog (MS) salts. **(A)** Shoot length of genotype Copenhagen Kush (CPH). **(B)** Shoot length of genotype Pennywise (PWE). **(C)** Leaf number of genotype Copenhagen Kush (CPH). **(D)** Leaf numbers of genotype Pennywise (PWE). The box of each dataset represents the interquartile range (IQR), which contains the third quartile (Q3–top side of the box), the median value of all of the data (the middle line), and the first quartile (Q1–bottom side of the box). The bars represent the maximum (Q3 + 1.5*IQR) and minimum (Q1–1.5*IQR) of the data. The numbers (*n*) above the bars depict explant numbers used.

Two basal salts media, Driver and Kuniyuki Walnut (cat.#D190; Phytotechnology Laboratories®, Lenexa, KS, United States) and Murashige and Skoog (cat.#M5524; Sigma Aldrich, Saint Louis, MO, United States), were compared for nodal explant growth using genotypes CPH and PWE. The composition of DKW medium, as used in this study, is as follows: 5.22 g/L DKW basal salts, 20 g/L sucrose, 0.1 g/L myo-inositol, 1 ml/L Gamborg B5 vitamins, 0.5 uM NAA, 1 uM TDZ, 1 g/L activated charcoal, and 3 g/L Phytagel. Explants were sterilized as described above but rinsed for 1 min instead of 3 min. They were transferred to either DKW or MM. Twenty jars, each containing a nodal explant, were placed under the lighting conditions described above. The initial height of the nodal explants was measured 4 days after placement on each medium to account for differences between initial explant size. The explants were transferred to the respective fresh media after 2 weeks, and 2 weeks later, the heights of the shoot from the base where it attached to the original explant to the top were measured using ImageJ. The height differences from the start to the end of the experiment represented the shoot height for each explant. The number of leaves on nodal explants on each medium was also assessed (Figure 3). The experiment was conducted twice. Only data from nodal stem segments that grew were included in the statistical analysis. The shoot height data for CPH on both media were compared by an unpaired two-sample *t*-test. For the data on leaf numbers for CPH and the shoot length data and number of leaves data for PWE, Wilcoxon Rank Sum non-parametric tests were performed as the data did not meet the “normality” assumptions of the unpaired two-sample *t*-test.

### Internal Contaminants in Nodal Explants

**Figure 4:**
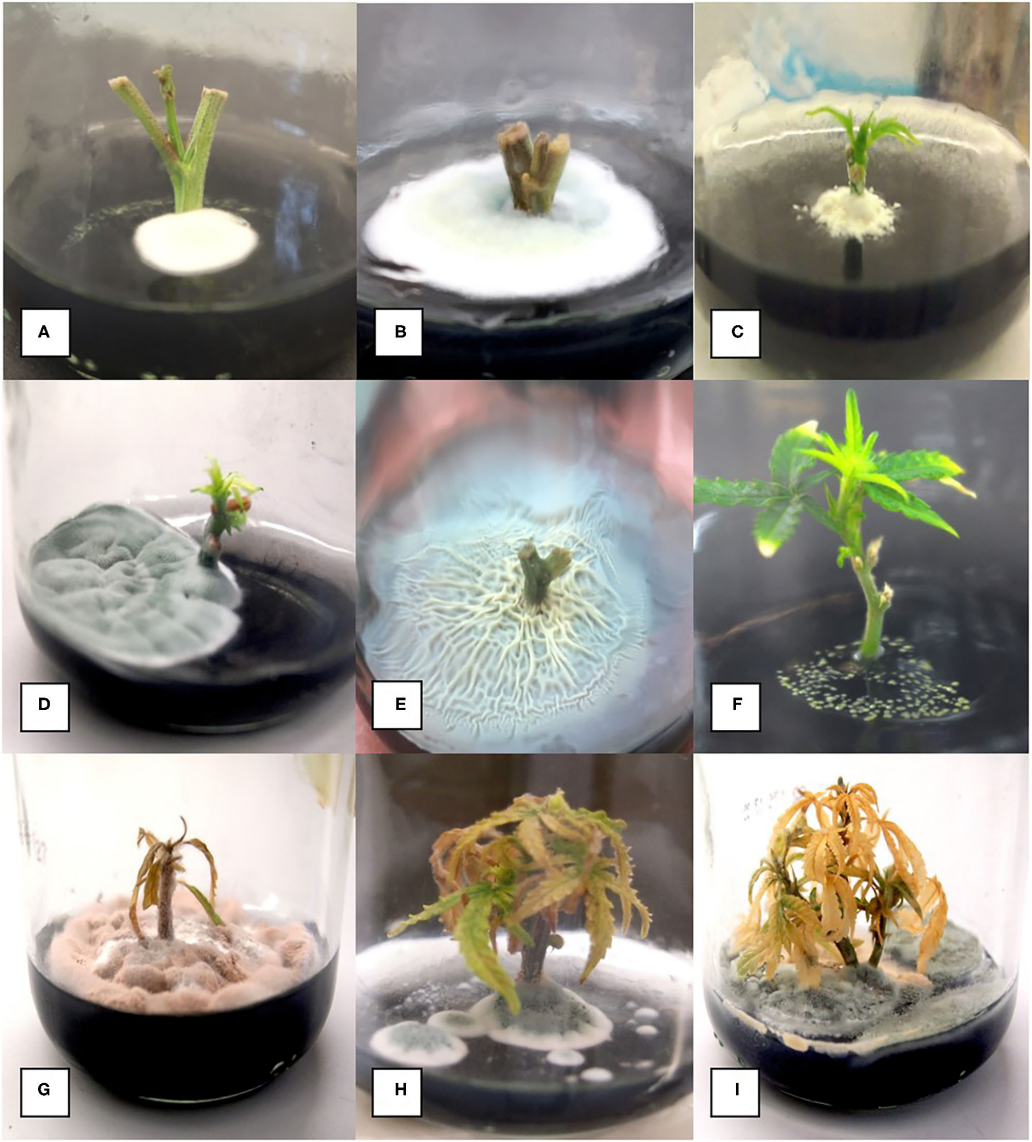
Range of microbial contaminants emerging from surface-sterilized nodal stem explants at various times after placement on MM containing activated charcoal. **(A)** Early emergence of *Penicillium* species. **(B)** Large colony of *Penicillium* growing from a nodal explant. **(C)** Colony of *Chaetomium* emerging from a nodal explant. **(D)** Large colony of *Penicillium* growing out of a nodal stem explant. **(E)** Extensive *Bacillus* growth emerging from a nodal stem explant. **(F)** Growth of *Pseudomonas* from a stem explant. **(G–I)** Death of established shoots from nodal stem segments due to contamination by microbes appearing later during plantlet growth.

In many nodal explants derived from donor plants used in tissue culture experiments, contamination by fungi, bacteria, and yeasts was frequently observed on the agar medium, in some cases up to 50%. This caused many of the explants to die (Figure 4). To reduce the level of microbial contamination, the following treatments were assessed by adding them to MM by filter sterilization after autoclaving: (1) the fungicide captan (Maestro 8-DF) was added at 0.01 and 0.02 g/L; (2) streptomycin sulfate was added at 100 mg/L; (3) Plant Preservative Mixture (PPM) (Plant Cell Technology, Washington, DC, United States) was added at 2 mg/L. For all treatments, 10 nodal stem segments of genotype CHQ were used for each of the treatments, and the experiment was conducted twice (*n* = 20). Assessments of the extent of microbial contamination were made after 4 weeks. To assess the effect of fungicides on endophytic contamination, the fungicide Luna (fluopyram) was applied to donor plants of genotypes CPH and PWE at 5 ml/L as a foliar spray until run-off. Plants were grown for 3 weeks before nodal stem segments were collected. Nodal stem segments from treated and control plants were dissected and sterilized with ethanol:bleach as described previously and rinsed with sterile distilled three times for 1 min each. The nodal stem segments were transferred to MM and grown under a 16-h photoperiod for 4 weeks, after which the proportion of jars with microbial contamination was evaluated. A total of 20 explants were included for each treatment group. The data obtained from the CPH genotype met the requirements for a chi-square test, but the data from PWE did not, so Fisher's Exact test was performed. The experiment was conducted once. The sample size was *n* = 20 for each group.

### Identification of Tissue Culture Contaminants

Colonies representing the most common contaminants seen in tissue culture were transferred to potato dextrose agar + streptomycin (130 mg/L) for 2 weeks and then to potato dextrose broth placed on a shaker at 125 rpm at room temperature for 7 days. The mycelium was harvested, and DNA was extracted using the DNeasy Plant Mini Kit (cat. No. 69104; QIAGEN, Hilden, Germany). For PCR, the internal transcribed spacer regions (ITS1 and ITS2) as well as the 5.8S gene of ribosomal rDNA were amplified using the universal eukaryotic primers UN-UP18S42 (5′-CGTAACAAGGTTTCCGTAGGTGAAC-3′) and UN-LO28S576B (5′-GTTTCTTTTCCTCCGCTTATTAATATG-3′) to produce a DNA template for sequencing. PCR conditions were as follows: initial denaturation at 94°C for 3 min, followed by 40 cycles of denaturation at 94°C for 30 s, annealing at 55°C for 45 s, and extension at 72°C for 2 min, and a final extension at 72°C for 7 min, followed by 4°C hold. PCR products were cut and sent to Eurofins Genomics (Eurofins MWG Operon LLC 2016, Louisville, KY, United States) for sequencing. The resulting sequences were compared with the corresponding ITS1-5.8S-ITS2 sequences from the National Center for Biotechnology Information (NCBI) GenBank database to confirm species identity using only sequence identity values above 99%. Once the identity of the cultured microbes was determined, random samples of nodal explants were obtained from the same donor plants (genotypes CPH and MBD), and 10–50 mg of fresh tissue was used for total DNA extraction and PCR following the conditions described above for fungal cultures. For examination of the nodal explants under a scanning electron microscope for potential microbes that could be present in internal tissues, samples were taken from donor plants known to be contaminated based on isolation in culture. They were processed following the procedure described by Punja et al. (2019).

### Rooting of Shoots From Nodal Explants

Genotype Moby Dick was used for root induction. Following the 8-week growth period for nodal segments, shoots measuring 2 cm in height were transferred to MS basal salts medium with the following amendments to induce rooting. The amendments were silver nitrate (40 μM), sodium metasilicate (6 and 9 mg/L), indole-3-butyric acid (5, 12.3, 37, and 42 μM), 2,4-dichlorophenoxyacetic acid (5 μM), and kinetin (1 μM). The shoots were incubated for 4 weeks on media containing these additives and rated for rooting frequency. Each treatment had a minimum of 10 explants, and the experiment was repeated three times (*n* = 30). The data were analyzed using Statplus version 2.21 and R systems (version 3.3.3). ANOVA was performed followed by Tukey's HSD test to determine significance at *P* < 0.05.

### Acclimatization for Plantlet Recovery

Shoots of genotype Moby Dick derived from either meristems or nodal explants that had formed roots were selected at random and transferred to one of the following growing substrates: peat plugs (Jiffy-7® peat pellets), rockwool cubes (2.5 × 2.5 × 3.8 cm, Grodan) or placed in a Turboklone (T-24 turbo-mini) (https://turboklone.com) containing a hydroponic nutrient solution with 1 ml/L of pH Perfect® Sensi Grow A&B and CALiMAGIc (General Hydroponics, Santa Rosa, CA, United States) and 0.25 ml/L of Rapid Start Rooting Enhancer (General Hydroponics, Santa Rosa, CA, United States) ~pH 5.8. Every 3 days, a nutrient mixture (without Rapid Start) was added to top up the system. The peat plugs/rockwool cubes were soaked for a minimum of 20 min in the same nutrient solution used for hydroponic growth. The plugs containing shoots were then placed in a sterilized tray (28 × 56 cm) and covered with a plastic dome (http://www.jiffypot.com/). The domes were misted with water every 2 days, and the vents were opened halfway after 7 days and fully opened after 9 days. Domes were removed 2 weeks post transfer. The percentage of survival of plantlets was assessed 4 weeks after transfer. Each treatment had a minimum of 10 replicates, and the experiment was conducted twice. The data were analyzed using Statplus version 2.21 and R systems (version 3.3.3). ANOVA was performed followed by Tukey's HSD test to determine significance at *P* < 0.05.

### Callus Growth

Genotypes Girl Scout Cookie, Space Queen, Moby Dick, and Pennywise were tested. After sterilization, leaf and petiole explants were placed on MM-AC. Petri dishes with leaf and petiole explants were kept on wire rack shelves under ambient conditions (temperature range of 21–25°C). One-half of the dishes were wrapped in aluminum foil to exclude light, and the remainder was left under the same conditions of supplemental lighting as for the nodal explants. Callus development was first observed after 4 weeks. Dishes were incubated for 6–10 weeks before measurements were taken. The percentage of explants that developed callus as well as the surface area of callus was measured. For the latter, an Alvin TD 1204 Circle Master Template (Alvin & Company, Bloomfield, CT, USA) was used to estimate the circle corresponding to the size that matched the callus, and the measurement was converted to cm^2^.

In a subsequent experiment, the following eight genotypes were compared: AFK, CPH, DEB, ISH, PWE, PNK, CBD, and WHR. Leaf segments from greenhouse-grown plants were sterilized in the bleach solution for 15 min and rinsed 3 times for 1 min in sterile ddH_2_O then placed on MM-AC. The dishes were wrapped in Al foil and incubated at 21–23°C for a maximum of 8 weeks. The callus area was measured using the “freehand” tool in ImageJ from photos taken of the Petri dishes that included a ruler for measurement. If the data did not meet the assumptions of parametric tests (i.e., ANOVA, unpaired two-sample *t*-test, etc.), then non-parametric tests were used (i.e., Welch's ANOVA, and Wilcoxon Rank Sum test). If the data met the assumptions of the ANOVA test, then they were used followed *post-hoc* by Tukey's HSD test.

## Results

### Shoot Growth From Meristem and Nodal Explants

Placement of meristems on MM medium (with 1% activated charcoal) resulted in shoot growth after 4 weeks in culture (Figure 1B), which continued to elongate after 8–10 weeks (Figures 1C,D). Baby food jars containing meristem explants at different stages of development were placed in a controlled environment growth chamber to allow for a comparison of growth of different genotypes (Figure 1E). Meristems of five different cannabis genotypes were assessed for their shoot regeneration response. After 10 weeks of growth, shoot height, number of axillary buds, and number of axillary shoots were measured (Table 1). The genotype with the greatest shoot height value was MBD, with an average height of 4.5 cm, which was significantly greater compared with genotypes CHQ, SPQ and PWE (*P* < 0.001). The average shoot growth across genotypes was 2.92 cm (Table 1). The number of axillary buds present on the shoots from meristems ranged from 8.5 for CBD to 3.4 for SPQ. The number of axillary buds produced per plantlet was not significantly different (*P* > 0.05) among CBD, CHQ, MBD, and PWE. SPQ had the lowest number of buds compared with the other genotypes (*P* < 0.05). Across genotypes, plantlets from meristems produced 6.38 axillary buds on average and 1.4 shoots (Table 1). Genotype SPQ showed poor growth for all parameters compared with the four other genotypes. While CBD plantlets were generally shorter, they had a higher number of buds that resulted in a bushier appearance. Genotypes MBD and PWE had similar shoot growth, but MBD was significantly taller than PWE (Table 1). Genotype SPQ generally showed the poorest growth among all the genotypes.

Measurements of height, number of axillary buds, and number of axillary shoots were obtained from nodal stem explants after 6 weeks (Table 1). The genotypes tested were BLD, SWD, MBD, and SPQ. The average shoot height ranged from 1.5 to 4.9 cm for SPQ and BLD, respectively (Table 1). BLD and SWD were significantly (*P* < 0.001) taller than MBD and SPQ at 4.9 and 3.8 cm, respectively. The average height across genotypes after 4 weeks was 3.075 cm. The average number of axillary buds across strains was 2.98, and the average number of shoots across genotypes was 0.3 (Table 1).

### Comparison of DKW and MS Salts

Shoot length and leaf numbers from nodal stem segments were evaluated for genotypes CPH and PWE after 4 weeks of growth on DKW or MM salts (Figure 3). For CPH, 78.4% of explants on DKW and 89.2% on MM basal salts grew and were included in a statistical comparison by an unpaired two-sample *t*-test. For PWE, 70% of explants on DKW and 77.5% on MM grew and were included in a statistical comparison by the Wilcoxon Rank Sum test. The data showed that there was a significant difference between DKW and MS basal salts in shoot length for CPH (*p* = 0.02947; Figure 3A). The average CPH shoot length on DKW and MM was 6.44 and 8.63 mm, respectively. In comparing shoot length for PWE on DKW and MM, there was no significant difference (*p* = 0.08354). The average PWE shoot length on DKW and MS was 7.66 and 9.2 mm, respectively (Figure 3B). For CPH leaf number, there was no difference between DKW and MM (*p* = 0.6882; Figure 3C). The average number of leaves on DKW and MM was 3.31 and 3.12, respectively. For PWE leaf number, there was a significant difference between DKW and MM (*p* = 0.04468; Figure 3D). The average number of leaves on DKW and MM was 4.13 and 5.64, respectively.

### Internal Contaminants and Alternative Sterilization Methods

In general, an average of 50% of nodal stem explants were found to be contaminated by microbes (a range of 10 to 80%), despite the rigorous sterilization methods used, and these had to be discarded. These contaminants included fungi, bacteria, and yeasts (Figure 4). Due to the high frequency of microbial contaminants from nodal stem explants when grown in a tissue culture medium, the addition of fungicides and antibiotics was assessed. The addition of a fungicide (captan) at 0.01 and 0.02 g/L to the tissue culture medium post-sterilization did not significantly reduce the contamination frequency on genotype CHQ (*P* = 0.05). The addition of streptomycin sulfate at 100 mg/L caused stunting and chlorosis of the tissue culture shoots (data not shown). Plant preservative mixture (PPM) at 2 ml/L appeared to delay the onset of contamination, but microbial growth would appear 1 to 3 weeks later. Nodal stem explants sterilized with 5% PPM + 3x basal salt solution or 70% EtOH and 10% bleach +0.1% Tween 20 showed no differences in microbial contamination on genotype CPH (60.5 vs. 60%, chi-square test *p* > 0.05). For CPH donor plants treated with Luna fungicide, a chi-square test was significant at *p* < 0.05, which suggests that fungicide treatment of donor plants could reduce the endophytic contamination observed in tissue culture. The CPH control group showed 88.2% contamination, and the Luna treatment group showed 31.8% contamination. However, the PWE plants treated with Luna did not differ significantly from the untreated control as overall contamination rates were much lower (12.5 vs. 16.7%, Fisher's exact test *p* > 0.05). These results indicate that genotypes may differ in terms of the extent of microbial contamination.

### Identification of Tissue Culture Contaminants

**Figure 5:**
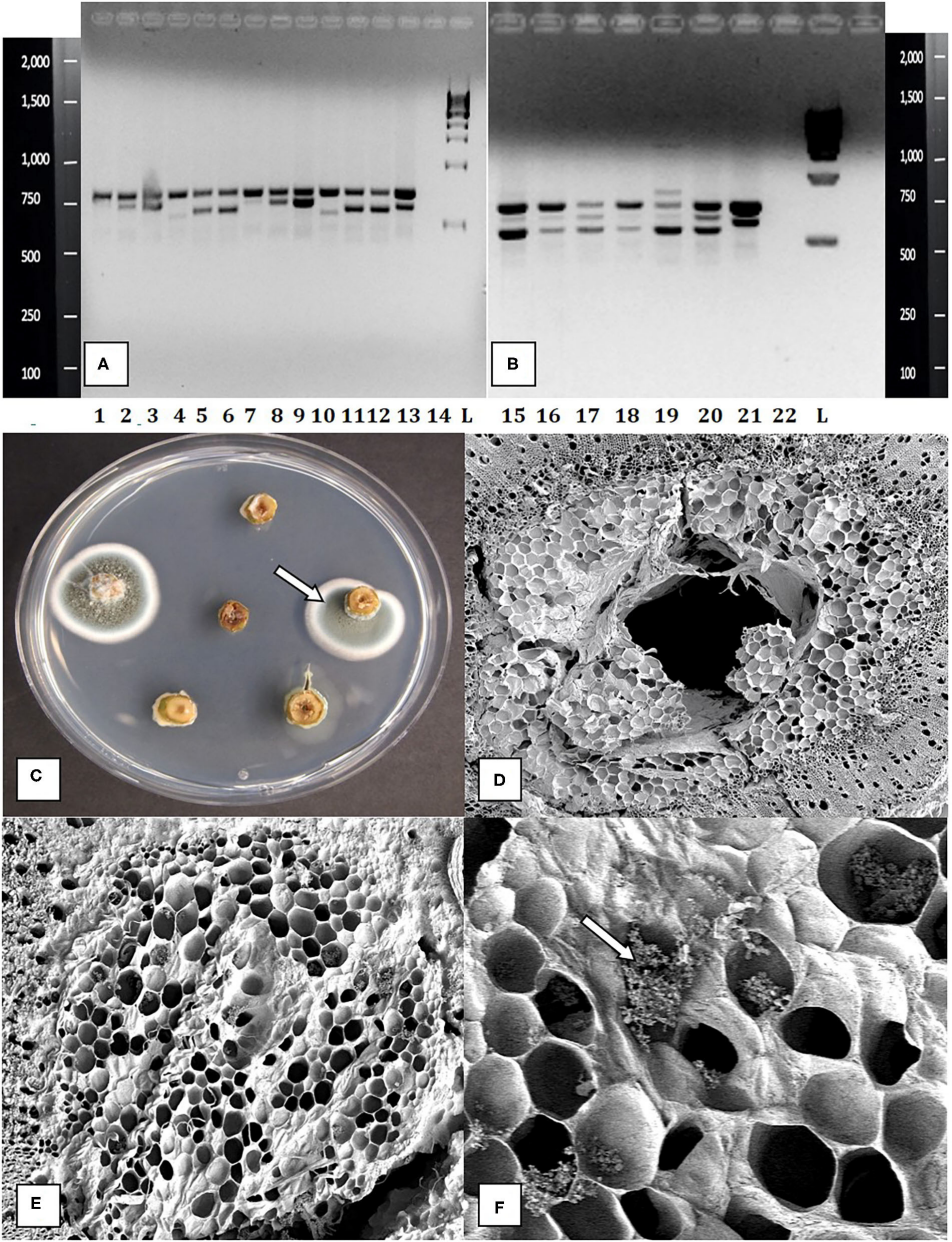
Molecular detection and identification of tissue culture contaminants. **(A,B)** PCR detection of fungal DNA present in naturally contaminated nodal explants. Upper bands at 750 bp size are plant DNA. Lower bands at ca. 650 bp are fungal DNA. These bands were cut and sequenced to determine the corresponding fungus present. Lanes 1–13 (lower bands) are as follows: clean, *Simplicillium lasoniveum, Trichoderma harzianum*. Clean, *Beauveria bassiana, Fusarium oxysporum*. Clean, *Trametes versicolor, Lecanicillium fungicola, Chaetomium globosum, F. oxysporum, F. oxysporum, L. fungicola*. Lane 14 = water control. L = molecular weight standards. Lanes 15–21 (lower bands) = *Penicillium chrysogenum, Penicillium copticola, C. globosum, Penicillium olsonii, P. olsonii, T. versicolor*. Lane 22 = water control, L= molecular weight standards. **(C)** Growth of *Penicillium* colonies emerging from the center of pith tissues in cut nodal segments. **(D)** Cross-section of a cannabis nodal stem explant showing the central pith surrounded by pith cells. **(E)** Close-up of pith cells as viewed in the scanning electron microscope. **(F)** Magnified view of pith cells in the scanning electron microscope showing fungal sporulation inside pith cells, likely of *Penicillium* sp. (arrow).

The contaminants observed growing on agar media consisted of bacteria, which were identified to genus level as *Bacillus* and *Pseudomonas*, yeasts that were not identified, and many fungi. In an attempt to better categorize the fungal contaminants, which represented the majority of contaminants present in nodal stem tissues, a PCR-based method was utilized. Cultures of 11 fungi recovered from nodal explants each produced a band size of about 650 bp after PCR. After sequencing and comparing the results in BlastN, a range of fungi were identified. This was followed by using naturally contaminated nodal explants, in which the frequency of detection of fungal contamination was 16/19 samples (Figures 5A,B). The plant DNA band was at 750 bp, as the universal primers used amplified both fungal and plant DNA. The genus and species of fungi present in cannabis stem tissues are shown in Figure 5. The PCR test could detect fungal contaminants present at DNA concentrations of 1 ng/ul (qPCR data not shown). Sections of stem segments when plated onto potato dextrose agar yielded colonies of *Penicillium* that emerged directly from the pith tissues (Figure 5C). When pith tissues were examined under a scanning electron microscope (Figure 5D), a close-up showed that fungal spores could be seen growing in and around pith cells (Figures 5E,F).

### Rooting of Shoots From Nodal Explants

**Figure 6:**
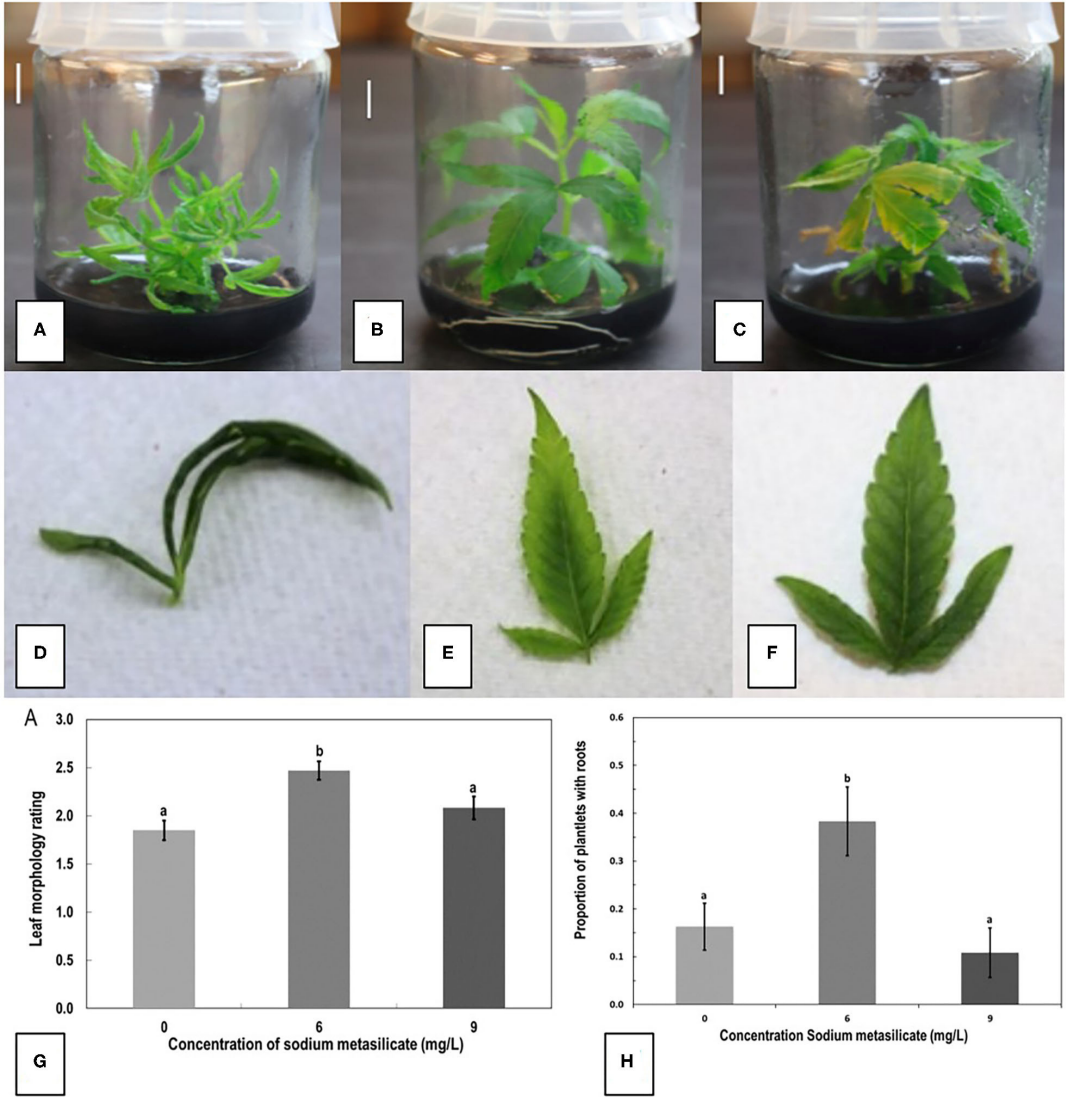
The effect of sodium metasilicate at 0 **(A)**, 6 **(B)**, and 9 **(C)** mg/L in the MM on shoot growth of genotype MBD from nodal segments. Optimal growth and rooting can be seen on 6 mg/L. **(D–F)** Leaf morphology rating scale applied to plantlets during growth in tissue culture as an indication of plantlet health. **(D)** A rating of 1 shows thin curled leaves, light green in color. **(E)** A rating of 2 shows leaves light green in color with some curling. **(F)** A rating of 3 shows dark green, flat leaves with serrated margins. **(G)** Average leaf morphology rating after 4 weeks of growth on MM with added sodium metasilicate. **(H)** Proportion of plantlets that developed roots on MM supplemented with sodium metasilicate at 6 mg/L was significantly different to the proportion of roots that developed from plantlets on MM, MM + sodium metasilicate 9 mg/L, and MMC. Bars followed by different letters indicate significant differences.

Representative plantlets from sodium metasilicate treatments of 0, 6, and 9 mg/L are shown in Figures 6A–C. The addition of sodium metasilicate caused visible differences in plant growth and leaf morphology, recorded on a scale of 1–3, as shown in Figures 6D–F. A rating of 1 shows thin curled leaves, while a rating of 3 shows dark green, flat, and toothed margins. The addition of sodium metasilicate at 6 mg/L significantly (*p* < 0.05) improved the leaf morphology rating compared with MM and MM + Na_2_SiO_3_ at 9 mg/L according to Tukey's HSD test (Figure 6G). On the MM + 6 mg/L Na_2_SiO_3_, the leaf morphology rating was comparable with the leaf rating of plants grown on the MM without Na_2_SiO_3_. The addition of Na_2_SiO_3_ at 6 mg/L produced the greatest proportion of rooted plantlets according to Tukey's HSD test (*p* < 0.05; Figure 6H). The proportion of rooted plants was 0.4 (40%) for MM + Na_2_SiO_3_ at 6 mg/L compared with 0.1, 0.1, and 0.2 for treatments MM, MM + 9 mg/L Na_2_SiO_3_, and MM without phytohormones (MMC). The addition of sodium metasilicate did not significantly affect any of the growth parameters measured for nodal stem segments or meristems: height, number of buds, and number of shoots (data not shown).

**Figure 7:**
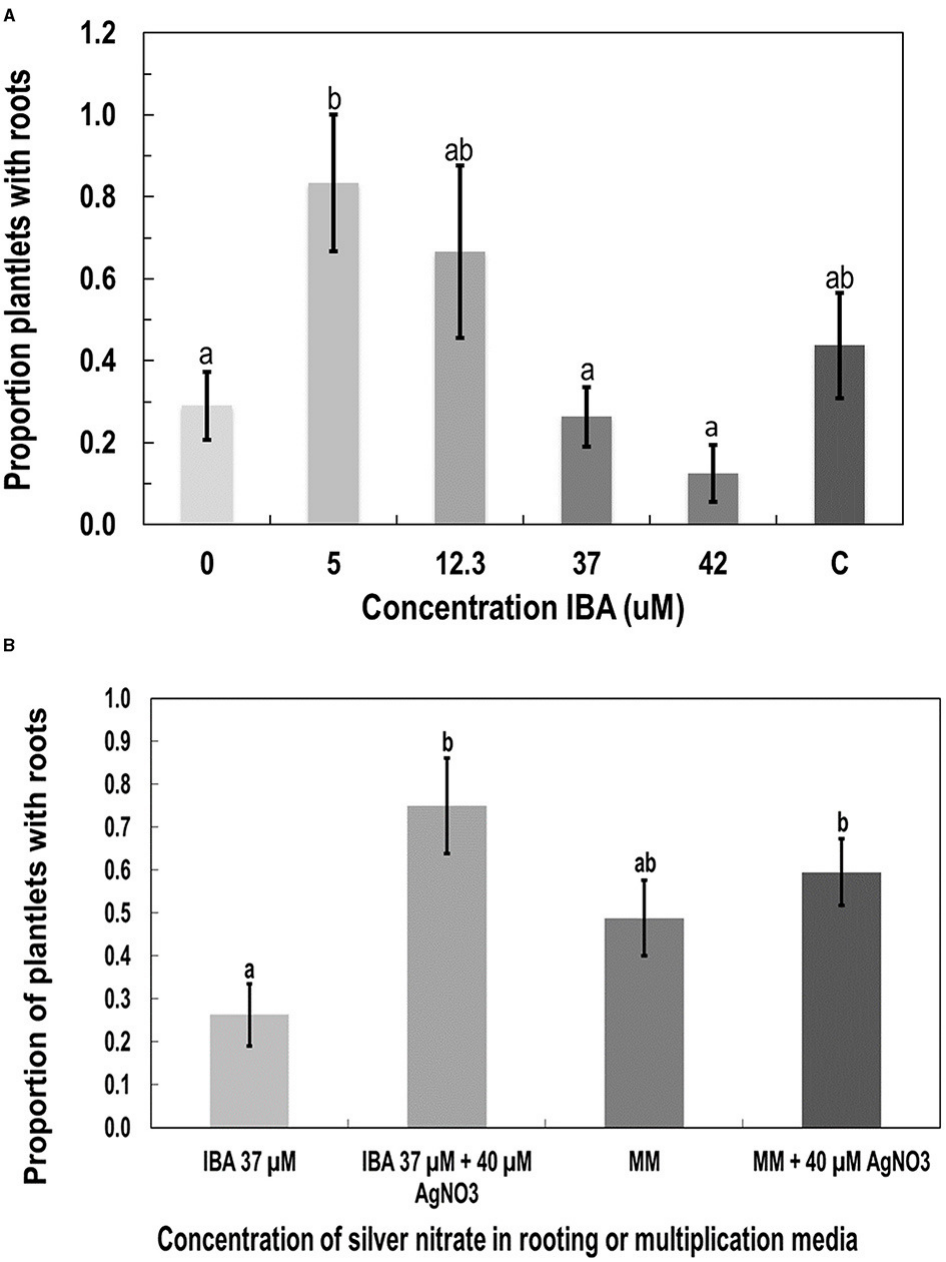
The effect of additives to the MS salts medium on root development in plantlets of genotype MBD derived from nodal stem segments after 4 weeks of growth. Additives were **(A)** indole-3-butyric acid IBA, **(B)** silver nitrate (AgNO_3_), compared with MM as a control. Statistical analysis was performed by Tukey's HSD test with significance at *p* < 0.05. Bars followed by different letters indicate significant differences.

The addition of indole-3-butyric acid, 2,4-dichlorophenoxyacetic acid (2,4-D), and kinetin (KIN) was tested as alternatives to TDZ and NAA in rooting media. KIN and 2,4-D were tested at 1 and 5 μM, respectively, alone and in combination. Neither hormone alone or in combination performed significantly better than the MM (data not shown). The combination of KIN and 2,4-D produced an average of 63% rooted plants, while the MM produced an average of 44% rooted plants. The MMC was used as the basal medium in the indole-3-butyric acid experiments. IBA was tested at 5, 12.3, 37, and 42 μM. While there was a trend toward decreased rooting as more IBA was added, the only statistically significant difference *(p* < 0.05 by Tukey's HSD test) was between 5 and 42 μM (Figure 7A). The MM (listed as C in Figure 7A) was not significantly different from any of the IBA concentrations; however, it averaged 44% rooted plants, while IBA at 5 μM produced 83% rooting.

Silver nitrate was added at 40 μM to the MM and MMC containing IBA at 37 μM instead of TDZ and NAA. The IBA with added silver nitrate had significantly more roots than IBA at 37 μM alone according to Tukey's HSD test (*p* < 0.05; Figure 7B). The AgNO_3_ did not significantly increase the proportion of roots produced when added to the MM.

### Rooting and Acclimatization for Plantlet Recovery

**Figure 8:**
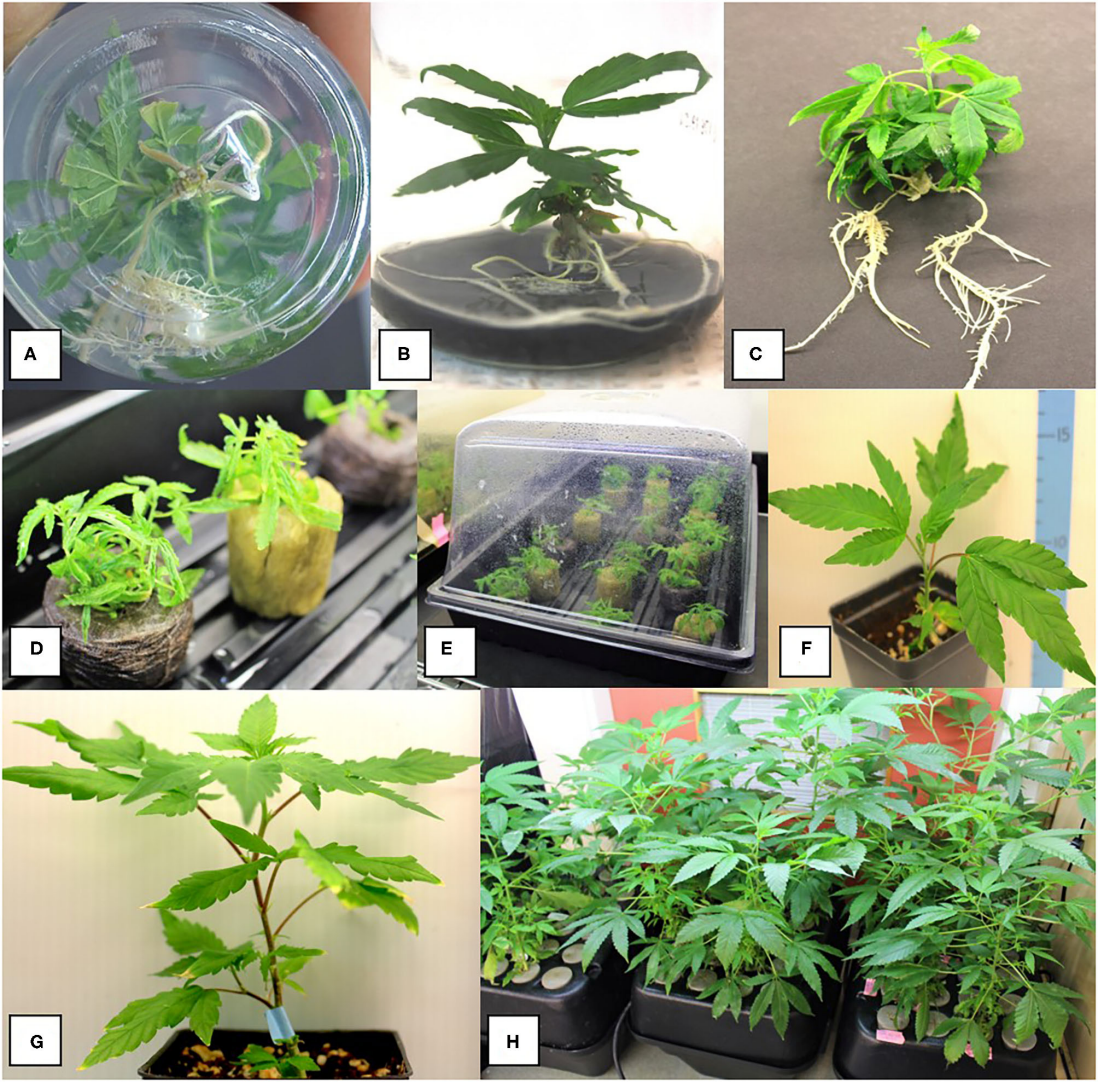
Root development on shoots after 8–10 weeks of growth in tissue culture and acclimatization to produce plants. **(A,B)** Spontaneous development of roots on nodal explants. **(C)** Plantlets were removed from tissue culture and transferred to coco growth media and placed under high humidity conditions for 2 weeks. **(D,E)** Acclimatization of plantlets from meristems of genotype MBD in different growth substrates. Plantlets were removed from tissue culture jars after 12 weeks of growth on the MM and placed into rockwool or peat under humid conditions for 14 days. Rockwool or peat plugs were soaked in a fertilizer mix of 1 ml/L of pH Perfect® Sensi Grow A&B and CALiMAGIc (General Hydroponics, Santa Rosa, CA, United States) in ~pH 5.8 water. **(F,G)** Growth of plants on a coco potting medium under ambient conditions following successful acclimatization. **(H)** Hydroponic system filled with 8 L of the fertilizer mix with vigorous growth of plants derived from meristem tissue cultures.

Spontaneous rooting was observed on some of the nodal explants (Figures 8A,B). Rooted plantlets were carefully removed from the tissue culture jars, rinsed of excess medium (Figure 8C), and transferred to a coco fiber growing medium. For comparison of rockwool, peat, or hydroponic system (Figures 8D,E), at least 10 plantlets were used per acclimatization substrate, and the experiment was repeated twice for different batches of plants (*n* = 20). The plantlets were of genotype MBD. Acclimatization success was calculated based on the number of healthy surviving plants after 2 weeks divided by the total number of plants that had been transferred for acclimatization. The survival success rate after acclimatization was 57, 76, and 83% for hydroponic, peat, and rockwool, respectively, which was not statistically different. Figures 8F,G show plants grown in coco growing substrate where they grew normally and produced more shoots. However, plants grown in rockwool appeared more vigorous and grew faster (Figure 8H)

### Callus Growth

**Figure 9:**
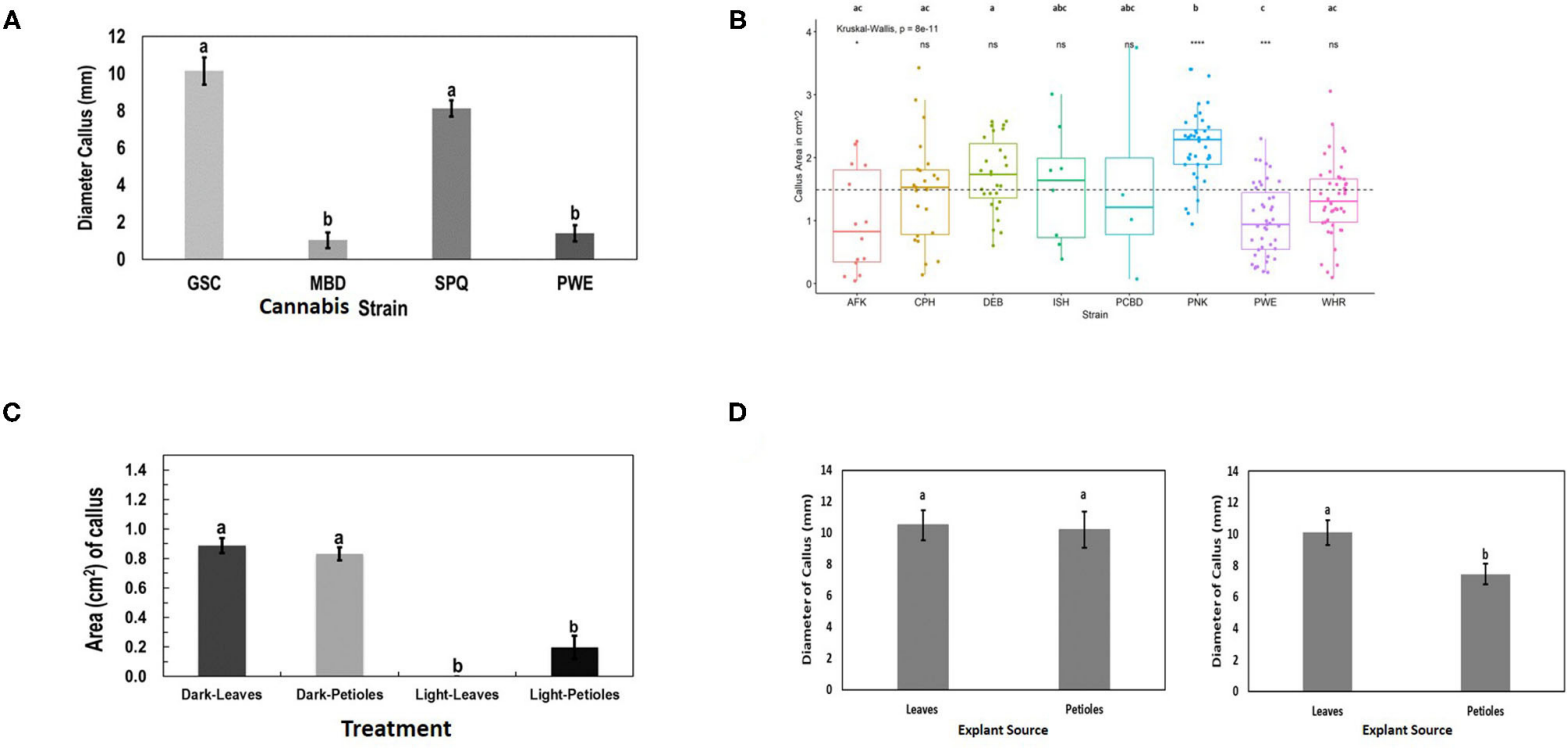
Response of cannabis genotypes to callus development on MM without activated charcoal (MM-AC). **(A)** Callus diameter of four genotypes developing from 1 cm^2^ leaf explants. **(B)** Callus area from leaf explants of 8 cannabis genotypes compared with the mean callus area across all strains (represented by the dotted line). The “*”represents significance level and “ns” represents not significant relationships between each genotype and across all genotype means (dotted line). A Kruskal–Wallis test resulting in a *p* of 8 × 10^−11^ and a Games–Howell *post-hoc* test were performed. Significant differences identified in the Games–Howell *post-hoc* test are shown using letters above the boxplots of each strain. **(C)** Growth of Cheesequake (CHQ) callus from leaf and petiole explants after 1 month under 24-h dark and 24-h light conditions on MM. Statistical analysis was performed by Tukey's HSD test with significance at *p* < 0.05. Bars followed by different letters indicate significant differences. **(D)** Growth of callus from leaves and petioles of genotype Girl Scout Cookie (GSC) (left) and Space Queen (SPQ) (right). Measurements of diameter were made after 4 weeks. Statistical analysis was performed by Tukey's HSD test with significance at *p* < 0.05. Bars followed by different letters indicate significant differences.

**Figure 10:**
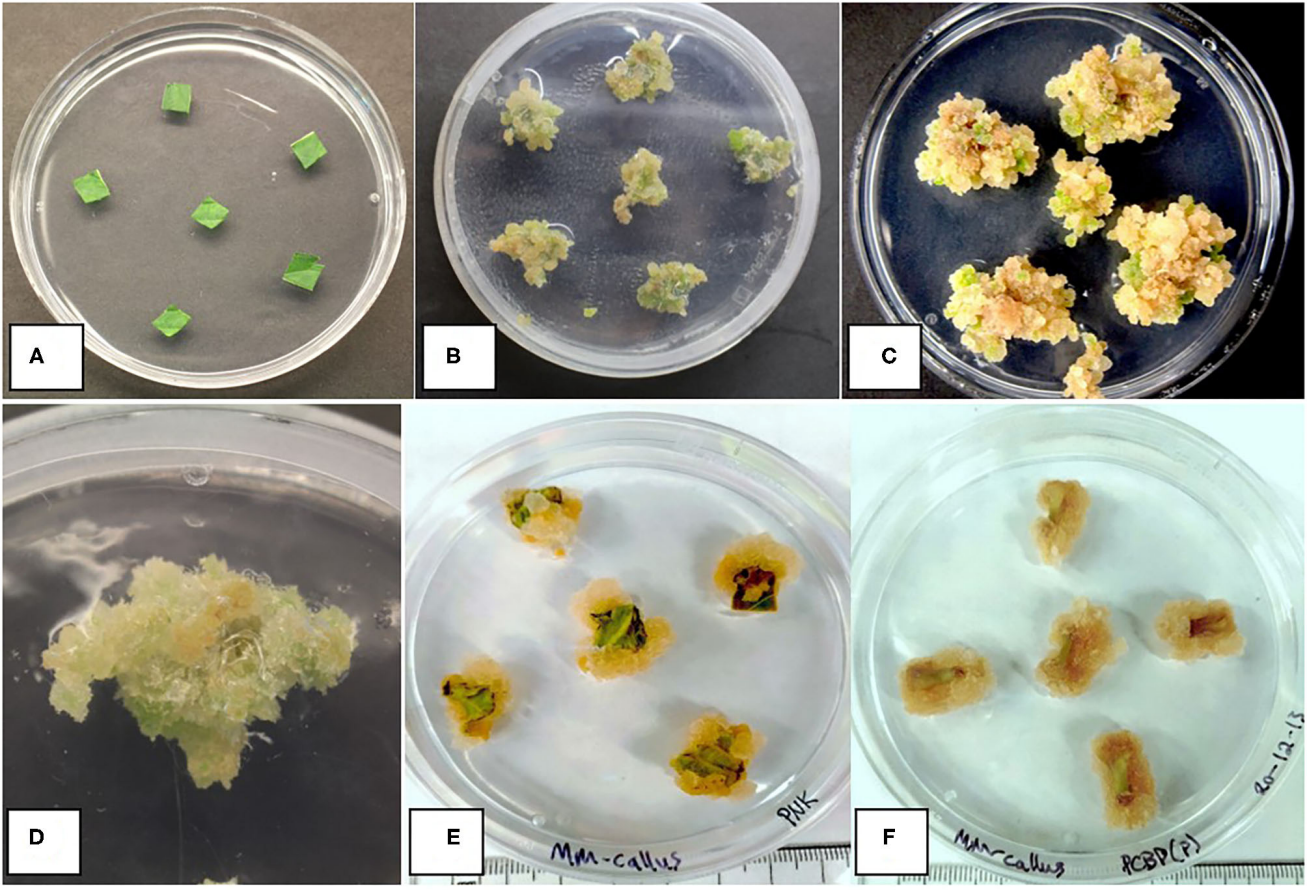
Callus development on leaf and petiole explants of different cannabis genotypes on MM-AC. **(A)** Leaf explants at the start of the experiment from a donor plant. **(B)** Callus from leaf explants of genotype SPQ after 8 weeks of growth. Variation in callus growth between explants can be seen. **(C)** Callus growth of genotype GSC from leaf explants after 8 weeks of growth. **(D)** Callus from leaf explants of genotype SPQ after 8 weeks of growth. **(E)** Callus from leaf explants of cannabis genotype Pink Kush after 8 weeks in the dark. **(F)** Callus from petiole explants of cannabis genotype Pure CBD after 8 weeks in the dark. No evidence of shoot development was observed in any of the callus cultures.

Callus development from leaves, measured as callus diameter, was genotype-dependent (Figure 9A). Genotypes GSC and SPQ readily developed callus, while for genotypes MBD and PWE, callus diameter was < 2 mm. In this experiment, one-way ANOVA was performed followed by Tukey's HSD test (*p* < 0.05). In the follow-up experiment, the mean callus area for the eight genotypes was compared by Welch's one-way ANOVA (*p* < 0.05). A Games–Howell non-parametric *post-hoc* test was then performed and identified that the following genotype comparisons were significantly different from one another: AFK vs. PNK, CPH vs. PNK, DEB vs. PNK, DEB vs. PWE, PNK vs. PWE, and PNK vs. WHR (Figure 9B). Leaf and petiole explants on media placed in the dark produced more callus than explants grown under light conditions (Figure 9C). Callus development from leaves and petioles showed genotype dependence. In SPQ, leaves developed more callus than petioles (*p* < 0.05), while on GSC, there was no significant difference between the explant types (*p* < 0.05; Figure 9D). The appearance of callus derived from leaf explants is shown in Figure 10. Within 4 to 8 weeks in culture, extensive callus growth could be observed from leaf segments (Figures 10A–D). The appearance of the callus was similar on leaf explants and petiole explants (Figures 10E,F). None of the calli showed evidence of differentiation toward shoot development or somatic embryo development. Further transfers of calli to new media eventually resulted in browning and death.

## Discussion

The results from this study conducted on drug-type cannabis (marijuana) show that the response to tissue culture conditions is first and foremost influenced by the genetic background (genotype) of the plants tested. The findings also indicated that both meristems and nodal explants were responsive to the tissue culture conditions tested, and that measurements of shoot growth could be used to determine the response of genotypes and quantify effects of media amendments on growth. Lastly, the findings show that the recovery of rooted plantlets is influenced by the degree of internal contamination of nodal explants and the extent to which rooting and acclimatization of the plantlets could be achieved using different treatments. Knowledge of these variables can enhance the successful recovery of plantlets from tissue cultures of *C. sativa*, which was the main objective of this study. This study focused on stage 1 of the micropropagation process as described by Page et al. (2021). This phase is equivalent to an initiation phase (establishment of cultures) and did not involve repeated cycles of subcultures and multiplication of shoots as observed in stage 2, the multiplication phase that increases plant numbers through micropropagation (Monthony et al., 2021; Page et al., 2021). Research on stage 1 is valuable to establish the genotypic response of a range of cannabis strains to initial tissue culture conditions and to rapidly recover plantlets from meristems or nodal explants for a first cycle of propagation.

Many previous reports of tissue culture of *Cannabis sativa* L. have utilized hemp varieties because of legal restrictions placed on the cultivation of drug-type cannabis in most regions of the world (Adhikary et al., 2021; Monthony et al., 2021). However, Lata et al. (2009) and Page et al. (2021) researched some of the variables that can influence the response of drug-type *C. sativa* to tissue culture conditions. In these studies, nodal segments with axillary buds were used for direct regeneration of shoots in stage 1 micropropagation (Lata et al., 2009, 2016; Page et al., 2021). The differential response of various genotypes to tissue culture conditions, as reported in this study, was also noted by Monthony et al. (2021). Prior research has shown that the response to tissue culture conditions in other plant species is affected by genotype (Islam et al., 2005; Martínez et al., 2017). If cannabis micropropagation is to be successful, an assessment of the response of genotypes of interest to tissue culture conditions would first need to be established before a full-scale tissue culture method could be developed for commercial use. In this study, some of the cannabis genotypes, e.g., MBD, produced shoots over 4.5 cm in height and formed multiple shoots and buds, while the other genotypes (SPQ and CHQ) grew poorly. Strain MBD was selected for a further study on plantlet recovery. A higher frequency of shoots and buds can potentially give rise to more plantlets during stage 2 micropropagation and can enhance plantlet numbers.

Meristems and axillary buds have both been used as starting explant sources for shoot induction in tissue culture experiments of various plant species. For example, axillary buds are commonly used for the propagation of fruit and nut trees, while meristems have been used for sweet potato and strawberry propagation (Hussain et al., 2012). Growth of axillary buds in tissue culture has been studied in mint (*Mentha* species) (Rech and Pires, 1986), Cancer tree (*Camptotheca acuminate*) (Liu and Li, 2001), hops (*Humulus lupus*) (Roy et al., 2001), Andean blueberry (*Vaccinium floribundum*) (Cobo et al., 2018), and other woody plants (Sahoo and Chand, 1998). In order to obtain plants free from pathogens, particularly viruses, meristem tip culture is a preferred method. Successful eradication of viruses using tissue culture techniques alongside a secondary method, such as thermotherapy, has been demonstrated in sugarcane (Cheong et al., 2012), *Lilium* spp. (Chinestra et al., 2015), dahlias (Nerway et al., 2020), artichoke (Spanò et al., 2018), and many others. We compared meristem and nodal explant types in this study and successfully obtained plantlets from both, with the meristems showing significantly lower microbial contamination rates compared with the nodal explants bearing axillary buds. However, shoot production from the meristems required a longer time in culture (10 weeks) compared with the axillary buds (6 weeks).

Meristems have not been previously tested as an explant source in either hemp or cannabis, although shoot tips were used for direct regeneration of hemp (Wang et al., 2009), and stem tips were used for micropropagation and retipping studies on hemp (Lubell-Brand et al., 2021). Meristems are important starting material, as they contain a lower frequency of internally-borne microbes and viruses (Wang and Hu, 1980). Since *C. sativa* L. is reported to harbor fungi and bacteria internally as endophytes (Scott et al., 2018; Punja et al., 2019), consequently, explants taken from mother plants that naturally carry endophytes or pathogens have a higher risk of becoming contaminated after transfer to tissue culture media, as observed in this study. Monthony et al. (2021) circumvented this problem by first establishing *in vitro* plants in stage 1 that were subsequently used to provide an explant material for studies on micropropagation and callogenesis in stage 2. While this approach is advantageous to provide clean explants and maintain desired genotypes *in vitro*, the explants used in the present study were derived from donor plants grown under commercial greenhouse conditions, as they provided unlimited quantities of tissues, and the plants could be evaluated for commercially desired traits prior to introduction into the tissue culture environment. To avoid higher contamination rates from these tissues, we evaluated a range of decontamination methods. Following reports of the addition of fungicides to tissue culture media to reduce fungal contamination (Nagy et al., 2005; Panathula et al., 2014), we added captan at 0.02 g/L, but it did not show any effect. We also tested a widely used broad spectrum product, Plant Presrvative Mixture™ (PPM; Plant Cell Technology, Washington, DC, United States), which reduced initial contamination in tissue culture but not beyond 2 weeks. Similarly, nodes that had been surface-sterilized in 5% PPM for 4 h showed no difference in contamination levels compared with nodes sterilized with 10% bleach +0.1% Tween 20. Interestingly, the application of a systemic fungicide (Luna) to the indoor-grown donor plants, followed 3 weeks later by the removal of nodal explants, showed reduced contamination levels in tissue cultures of one strain by almost 3-fold.

The contaminating microbes in cannabis explants appear to reside within the central pith tissues, as shown in this study using scanning electron microscopy and reported elsewhere (Punja et al., 2019). This could explain the difficulty in eradicating them with surface-sterilization methods. Donor plants of some genotypes, e.g., CPH appeared to have a higher background level of contamination compared with other genotypes, e.g., PWE. Most of the contaminants emerged after several weeks in the tissue culture environment and originated from the central pith of the nodal explants (see Figure 5). Axillary buds may contain pathogens or endophytes living internally, which can easily be transferred into tissue culture (Wang and Hu, 1980). Internal contamination is less of a concern for meristems, as the vascular dome and first primordial leaves are generally free of bacteria, fungi, and viruses (Ramgareeb et al., 2010). Previous studies have demonstrated that an extensive array of fungal and bacterial endophytes can colonize hemp and cannabis plants (Kusari et al., 2013; Scott et al., 2018; Punja et al., 2019). Kusari et al. (2013) found 30 different species of fungal endophytes, of which P*enicillium copticola* was the most prevalent. Scott et al. (2018) found 134 bacterial and 54 fungal strains in three hemp cultivars. The most abundant fungal genera were *Aureobasidium, Alternaria*, and *Cochliobolus*, and the most abundant bacterial genera were *Pseudomonas, Pantoea*, and *Bacillus*. Punja et al. (2019) showed that endophytic and pathogenic fungi, such as species of *Chaetomium, Trametes, Trichoderma, Penicillium*, and *Fusarium*, could colonize cannabis plants internally. Previous tissue culture studies on hemp and cannabis have not described problems with tissue culture contaminants. It is likely that the coco fiber used as a substrate for growing plants in this study harbored microbes that eventually colonized the internal tissues of the stems and became established (Punja et al., 2019). Other growing media, such as rockwool, may contain lower levels of contaminating microbes. Donor plants grown in a coco fiber substrate over prolonged periods of time in indoor environments, e.g., for up to a year, such as CPH, showed much higher background levels of contaminants. Recent microbiome studies have demonstrated that the bulk soil and rhizosphere of cannabis and hemp plants are the most influential in determining the subsequent composition of internal microbes in other regions of the plant (Barnett et al., 2020; Comeau et al., 2021). Therefore, attention should be given to the condition of donor plants with regard to the substrate they are grown in and their duration in the growing environment. Young plants grown in relatively sterile growth substrates should be selected for tissue culture studies.

A polymerase chain reaction-based assay showed conclusively that cannabis stem tissues contained a range of fungi. The method allowed the detection of 1 ng/ml of genomic DNA and could be used to screen donor plants to determine the background level of microbial contamination. Similar PCR-based methods have been used to screen mother plants and tissue-cultured plants such as strawberries, sweet potatoes, and roses to ensure they are free of bacteria and fungi (Moreno-Vázquez et al., 2014; University of California Davis, 2008). This approach can be applied to cannabis plants before they are deployed in tissue culture. In addition, if meristem culture of cannabis is used to obtain pathogen-free plantlets, it would have to be accompanied by a similar PCR-based assay to test for the absence of these pathogens. Nodal explant cultivation is unlikely to be free of pathogens given the high levels of internal contamination observed in this study. Therefore, shoots derived from nodal cultures should be avoided because of the potential for contaminants. Meristems represent the explant of choice to obtain pathogen-free plantlets from tissue cultures of *C. sativa*.

The tissue culture medium used for growth of plant tissues can influence the success in initiation and multiplication of shoot growth and elongation. Following shooting, a second medium with a higher concentration of auxin can be used to induce rooting (Lata et al., 2009; Wang et al., 2009; Chandra et al., 2017). We used the multiplication medium described by Lata et al. (2009) containing Murashige and Skoog (MS) basal salts supplemented with myo-inositol and activated charcoal. The growth regulators added were thidiazuron (TDZ, 1.0 μM) and naphthaleneacetic acid (NAA, 0.5 μM). On this medium, both explant types responded favorably, and shoots were produced in the initiation phase and could be transferred to subsequent media of the same composition for measurements to be made. However, continuous subcultures over extended time periods on MS salts medium tended to produce shoots that displayed hyperhydricity and developed signs of nutrient deficiency with low multiplication rates (authors, unpublished observations). These symptoms were also described by Page et al. (2021) on MS salts medium. The addition of activated charcoal appeared to improve growth on the MS medium; therefore, MS salts plus 1% activated charcoal was used in most of the experiments in this study. Activated charcoal, when added to tissue culture media, can absorb or bind some of the toxic waste compounds released from growing plants, in particular phenolic compounds, thereby improving *in vitro* plant tissue growth (Wang and Huang, 1976; Thomas, 2008; Chandra et al., 2017). This would be particularly useful in stage 1 micropropagation. Page et al. (2021) reported that a tissue culture medium based on DWK basal salts supported better canopy growth than MS basal salts from nodal explant segments that were intended for stage 2 micropropagation. They compared five cannabis genotypes and used two-node explants originating from micro-propagated plantlets grown on a DWK salts medium. Their results showed that explants grown on DKW produced a larger canopy area and had a higher multiplication rate than explants grown on MS. In this study, comparisons between the two basal salts media using two cannabis genotypes did not show consistent differences in shoot growth that could be attributed to the effect of medium composition during stage 1 micropropagation. Wang et al. (2009) used an MS basal salts medium with 30 g/L sucrose, 6.8 g/L phytagel, and 1 μM of TDZ to produce axillary buds from shoot tips of hemp during micropropagation. Lubell-Brand et al. (2021) used an MS salts medium described by Lata et al. (2016) in which TDZ was replaced with the growth regulator meta-topolin (mT). They reported that hyperhydricity was reduced by modifying the agar content of the medium, coupled with improved vessel ventilation and enhanced nitrogen levels. Therefore, both DKW and MS salts media can support short-term growth in tissue culture media during the initiation of cultures. However, continuous subcultures and multiplication on a DKW-based medium appear to yield healthier and more vigorous plants (Page et al., 2021) or on an MS medium supplemented with enhanced levels of nitrogen (Lubell-Brand et al., 2021).

Rooting is often the most challenging step in micropropagation (equivalent to Stage 3 micropropagation according to Page et al., 2021), especially in woody plant species (Ranaweera et al., 2013). IBA, a naturally occurring auxin, has previously been shown to induce rooting in cannabis plants at 5 μM (Lata et al., 2009), which was confirmed in this study. However, since the rooting frequency with IBA was not significantly different from that with MM, further rooting experiments with sodium metasilicate and silver nitrate were conducted on MM. KIN and 2,4-D were also tested for promotion of rooting. In callus cultures, these hormones induced rooting (Feeney and Punja, 2003). When added to MMC, neither KIN nor 2,4-D alone or in combination induced rooting in plantlets to the extent reported from callus. To promote root induction, sodium metasilicate (containing 22.9% silicon) was added to MM. Silicon was included because of its reported positive impact on rooting seen in other plant species (Zhuo, 1995; Soares et al., 2011). Previous research has also shown a positive effect of the addition of silicon to tissue culture media on leaf morphology of banana (*Musa acuminata*) (Luz et al., 2012). In this study, a significant increase in rooting and improved leaf morphology were observed when sodium metasilicate was added to MM at 6 mg/L. Sodium metasilicate has not been previously used in tissue culture of *C. sativa*. In this study, silver nitrate (AgNO_3_) increased the number of rooted plants when added with IBA, but had no significant effect when added to MM. AgNO_3_ could be combined with a lower concentration of IBA (5 instead of 37 μM) or with sodium metasilicate to determine the effects on plantlet recovery. However, the use of AgNO_3_ may alter the sex expression toward male flower formation (Punja and Holmes, 2020).

The final step in tissue culture propagation is acclimatization of plantlets (Stage 4). At this stage, plantlets are removed from the jars/containers and acclimatized to external environmental conditions. When removing plantlets from the medium, roots should be carefully washed to avoid damage, and the agar should be washed off to prevent fungal growth from the residual sucrose. In this study, plantlets were transferred directly to rockwool, peat, and hydroponic substrates for a comparison of survival following acclimatization. Rockwool, peat, and coco fiber are the most common soilless growing media used worldwide for the production of fruits, vegetables, and cut flowers (Savaas and Gruda, 2018). In the present study, the plantlets generally grew better in rockwool during acclimatization, followed by peat and the hydroponic system, although the substrate response was not statistically different. The plantlets exhibiting a bushy morphology with long thin curled leaves acclimatized poorly. The addition of sodium metasilicate improved the morphology of the plants and was a contributing factor to improved acclimatization. Lata et al. (2016) acclimatized and hardened well-rooted cannabis plantlets with a 100% survival rate by 10-day pre-incubation on a coconatural medium before transfer into potting mix-fertilome. A mixed approach of using tissue culture medium with sterile rockwool cubes for multiplication and rooting of cannabis (Kodym and Leeb, 2019) may be a good option for improving acclimatization. Rooting can also be done *ex vitro*, i.e., outside the tissue culture environment. For example, a peat-based medium and high humidity conditions were used successfully for tea plants (*Camellia sinensis* L.) (Ranaweera et al., 2013). When compared with conventionally propagated tea plants using tissue culture, the *ex vitro* rooted micro shoots produced superior plants. Similarly, an *in vitro*–*ex vitro* micropropagation system was recently described for hemp (Lubell-Brand et al., 2021).

In addition to direct regeneration of shoots from axillary buds or meristems, efforts have been made toward indirect regeneration of shoots from callus cultures of hemp and cannabis. These have not been successful because of the recalcitrant nature of this species (Monthony et al., 2021). Differences in callus growth from petioles and leaves were attributed to different genetic backgrounds of the plants tested. Slusarkiewicz-Jarzina et al. (2005) and Wielgus et al. (2008) studied the effect of plant growth regulators on the development of callus and subsequent regeneration in five hemp genotypes. Their results showed that genotype was an important and determining factor for callus growth and regeneration. The hemp cultivar Fibrimon-24 produced the most calluses (83%), while a different cultivar, Silesia, had a regeneration rate of only 2.5%. In previous studies, callus formation has been induced from both hemp and cannabis explant tissues using combinations of the auxins 2,4-dichlorophenoxyacetic acid (2,4-D), naphthaleneacetic acid (NAA), and indole-3-butyric acid (IBA), and the cytokinins kinetin (KIN) and thidiazuron (TDZ) (Braemer and Paris, 1987; Feeney and Punja, 2003; Lata et al., 2009; Wahby et al., 2013; Movahedi et al., 2015). Various explants have been studied for callus induction in hemp and cannabis, such as cotyledons and epicotyls (Wielgus et al., 2008; Movahedi et al., 2015), leaves (Mandolino and Ranalli, 1999; Page et al., 2021), and petioles (Slusarkiewicz-Jarzina et al., 2005). In this study and in that of Page et al. (2021), the genotype was shown to influence the extent of callus formation. Page et al. (2021) found that the addition of 2,4-dichlorophenoxyacetic acid to media was required for callus production, and that media containing DWK salts yielded healthier and faster-growing calluses than the MS medium. We did not test callus growth on the DWK salts medium. Interestingly, genotype SPQ, which responded poorly for shoot growth from meristems and nodal explants, responded well to callus production in this study. In contrast, genotype MDB, which responded very well to shoot growth, produced the least callus. The interest in deriving calluses from cannabis explants followed by regeneration of shoots is to allow genetic transformation studies to succeed (Feeney and Punja, 2003). In addition, there are numerous applications of tissue culture methods for cannabis and hemp improvement, and these have been described elsewhere (Adhikary et al., 2021). To date, however, there are few reports describing the successful utility of tissue culture approaches for *C. sativa* on a large and cost-effective scale.

The interest in micropropagation through tissue culture is to produce genetically identical, pathogen-free plants year-round in a limited amount of space (Yancheva and Kondakova, 2018; Lubell-Brand et al., 2021; Monthony et al., 2021). The results of this study, and those of previous investigators (Lata et al., 2009; Wang et al., 2009; Page et al., 2021) show that it is possible to obtain plantlets from tissue cultures of drug-type *C. sativa*, but the process requires a detailed assessment of the genotypic response, evaluation of the effect of basal salts medium, establishment of conditions promoting shoot growth and rooting frequency, and achievement of success in acclimatization. In addition, distinguishing between the requirements of stage 1 micropropagation (initiation of tissue cultures) verses stage 2 micropropagation (multiplication of shoots), as pointed out by Page et al. (2021), may result in differing protocols being developed. In contrast to the vegetative clonal propagation method that is widely used in the cannabis industry, tissue culture approaches will still play a minor role in commercial production until research efforts have resolved many of the remaining challenges and the cost-effectiveness of this approach is proven. This study has attempted to assess the variables that can affect success in plantlet recovery from stage 1 micropropagation using meristems and nodal stem explants in order to provide future directions in this area.

## Data Availability Statement

The raw data supporting the conclusions of this article will be made available by the authors, without undue reservation.

## Author Contributions

All the authors contributed in the formulation of concepts of the research and designed the experiments, collected the data, wrote the manuscript, and prepared the figures.

## Funding

Funding was provided through a Collaborative Research and Development (CRD) grant from Agrima Botanicals and the Natural Sciences and Engineering Research Council of Canada (NSERC).

## Conflict of Interest

The authors declare that the research was conducted in the absence of any commercial or financial relationships that could be construed as a potential conflict of interest.

## Publisher's Note

All claims expressed in this article are solely those of the authors and do not necessarily represent those of their affiliated organizations, or those of the publisher, the editors and the reviewers. Any product that may be evaluated in this article, or claim that may be made by its manufacturer, is not guaranteed or endorsed by the publisher.
